# Supplementary material for: PD-1- CD45RA+ effector-memory CD8 T cells and CXCL10+ macrophages are associated with response to atezolizumab plus bevacizumab in advanced hepatocellular carcinoma
Source: Nat Commun. 2023 Nov 29;14:7825. doi: 10.1038/s41467-023-43381-1 (PMC10687033; doi:10.1038/s41467-023-43381-1)
Supplement: Supplementary file 1 — Supplementary Information [file 41467_2023_43381_MOESM1_ESM.pdf]

PD-1^{-} CD45RA^{+} effector-memory CD8 T-cells and CXCL10 macrophages are associated with response to atezolizumab plus bevacizumab in advanced hepatocellular carcinoma

Sarah Cappuyns[1,2,3,4], Gino Philips[3,4], Vincent Vandecaveye[5,6], Bram Boeckx[3,4], Rogier Schepers[3,4], Thomas van Brussel[3,4], Ingrid Arijs[3,4], Aurelie Mechels[3,4], Ayse Bassez[3,4], Francesca Lodi[3,4], Joris Jaekers[7], Halit Topal[7], Baki Topal[7], Orian Bricard[3,4], Junbin Qian[3,4,8,9], Eric Van Cutsem[1,2], Chris Verslype[1,2], Diether Lambrechts[3,4,*], Jeroen Dekervel[1,2,*]

[1]Digestive Oncology, Department of Gastroenterology, University Hospitals Leuven, Leuven, Belgium

[2]Laboratory of Clinical Digestive Oncology, Department of Oncology, KU Leuven, Leuven, Belgium

[3]Laboratory for Translational Genetics, Department of Human Genetics, KU Leuven, Leuven, Belgium

[4]VIB Centre for Cancer Biology, Leuven, Belgium

[5]Radiology Department, University Hospitals Leuven, Leuven, Belgium

[6]Laboratory of Translational MRI, Department of Imaging and Pathology, KU Leuven, Leuven, Belgium

[7]Hepatobiliary- and pancreas Surgery, Department of Abdominal Surgery, University Hospitals Leuven, Leuven, Belgium

[8]Zhejiang Provincial Key Laboratory of Precision Diagnosis and Therapy for Major Gynaecological Diseases, Women's Hospital, Zhejiang University School of Medicine, Hangzhou, China

[9]Institute of Genetics, Zhejiang University School of Medicine, Hangzhou, China

Supplementary Data

1. **Supplementary Figures S1-S7**
2. **Supplementary Tables S1-S3**

1. Supplementary Figures

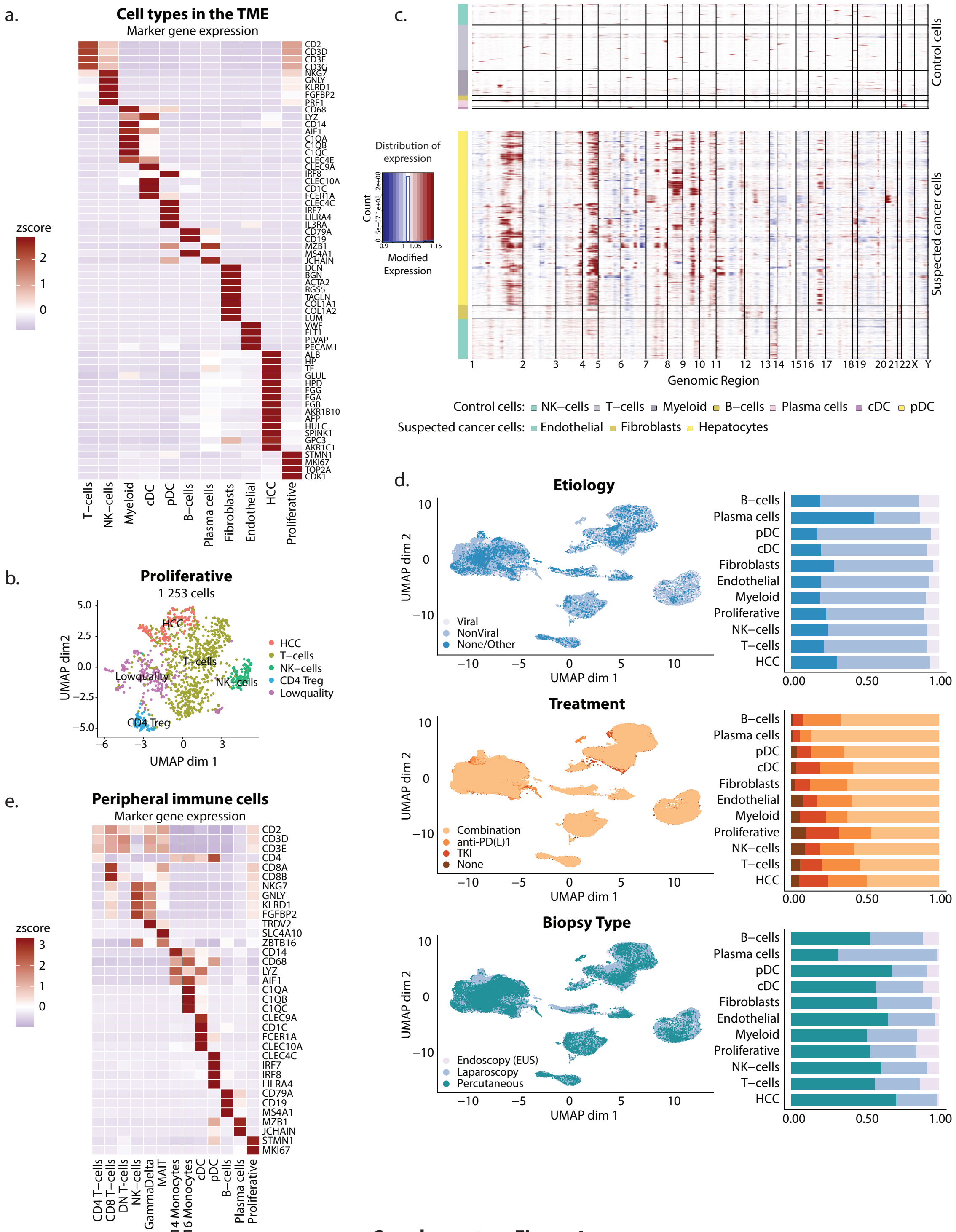

Supplementary Figure 1

Supplementary Figure 1. **The TME and peripheral immune system of aHCC**

a. Heatmap depicting the expression of marker genes in each cell type identified in the TME of aHCC. **b.** UMAP representation of the 'Proliferative' cluster, consisting of mostly proliferating T-cells. **c.** Inferring CNV from scRNAseq data. Top: CNV profiles of non-malignant controls: NK-cells, T-cells, Myeloid, B-cells, Plasma cells, cDC and pDC. Bottom: CNV profiles of suspected cancer cells. **d.** UMAP and bar plots colour-coded for underlying liver disease (*top)*, treatment (*middle*) and biopsy type (*bottom*), showing their distribution across cell types. **e.** Heatmap depicting the expression of marker genes in each cell type identified in peripheral blood.

(CNV, Copy number variations; cDC, Conventional denritic cells; pDC, Plasmacytoid dendritic cells; DN T-cells; Double Negative T-cells; EUS, Endoscopic ultra-sound; MAIT, Mucosal-associated invariant T-cells; TKI, tyrosine kinase inhibitor; TME, Tumour-microenvironment; UMAP, Uniform Manifold Approximation and Projection).

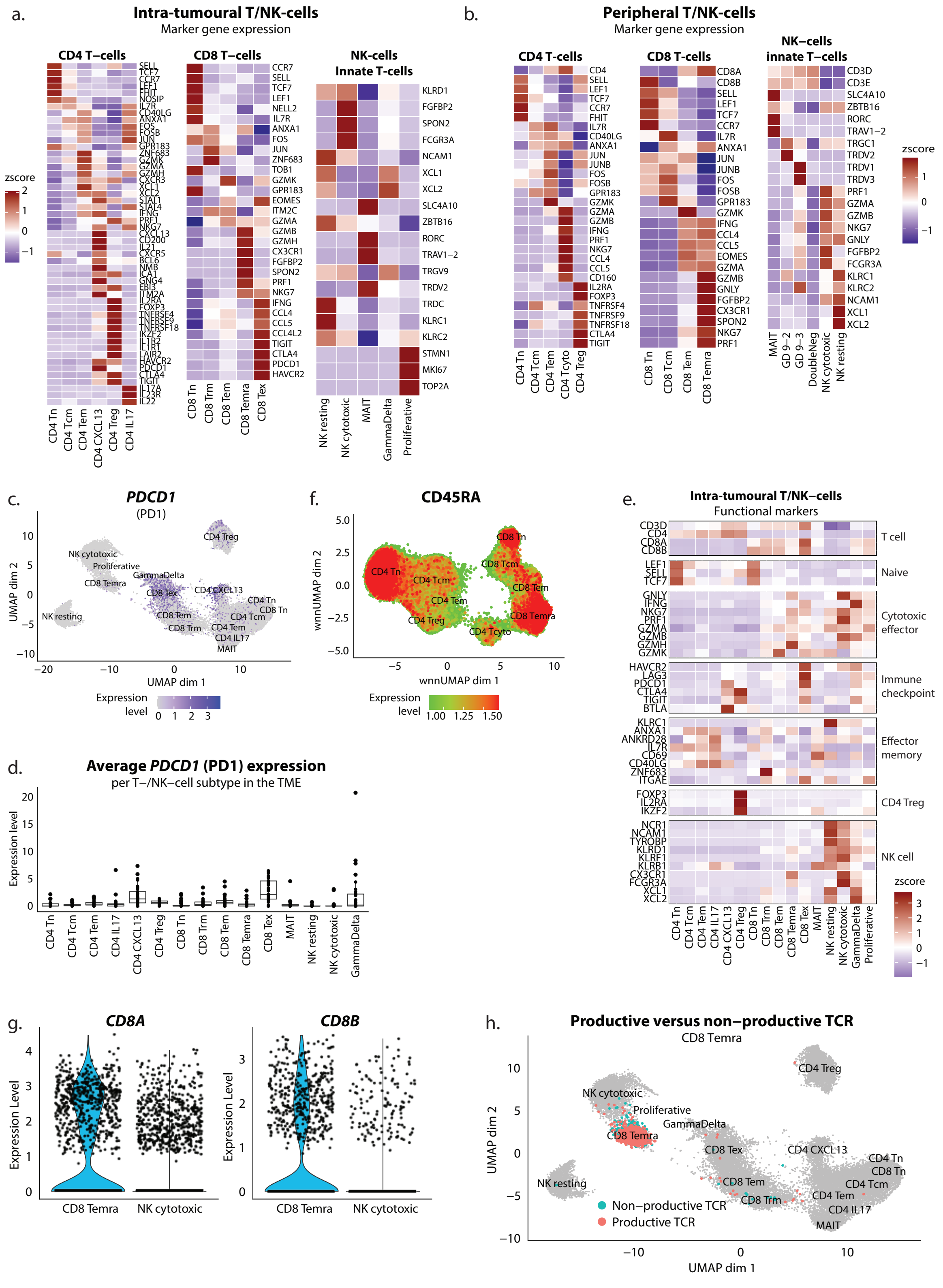

Supplementary Figure 2

Supplementary Figure 2. **Subclustering and annotation of intra-tumoural and peripheral T/NK-cell phenotypes**

a. Heatmaps showing expression of marker genes used for annotation of intra-tumoural T/NK-cell phenotypes. **b.** Heatmaps of showing the expression of marker genes used for annotation of peripheral T/NK-cell phenotypes. **c.** Feature plot of *PDCD1* (PD1) expression in intra-tumoural T/NK-cells. **d.** Boxplots depicting average *PDCD1* (PD1) expression level, calculated per patient (n=38) in each intra-tumoural T/NK-cell phenotype. Boxes indicate median +/- interquartile range; whiskers show minima and maxima. **e.** Heatmap displaying expression of functional genes in intra-tumoural T/NK-cell phenotypes. **f.** Feature plot of CD45RA using TotalSeq-C data in peripheral T-cells. **g.** Violin plot of *CD8A (*left) and *CD8B (right)* expression in intra-tumoural CD8 T_{EMRA} and $NK_{cytotoxic}$. **h.** UMAP representation of intra-tumoural T/NK-cells, coloured for productive versus non-productive TCR sequences in CD8 T_{EMRA} cells.

(GD, Gamma-delta T-cells; PD1, Programmed cell death protein 1; UMAP, Uniform Manifold Approximation and Projection).

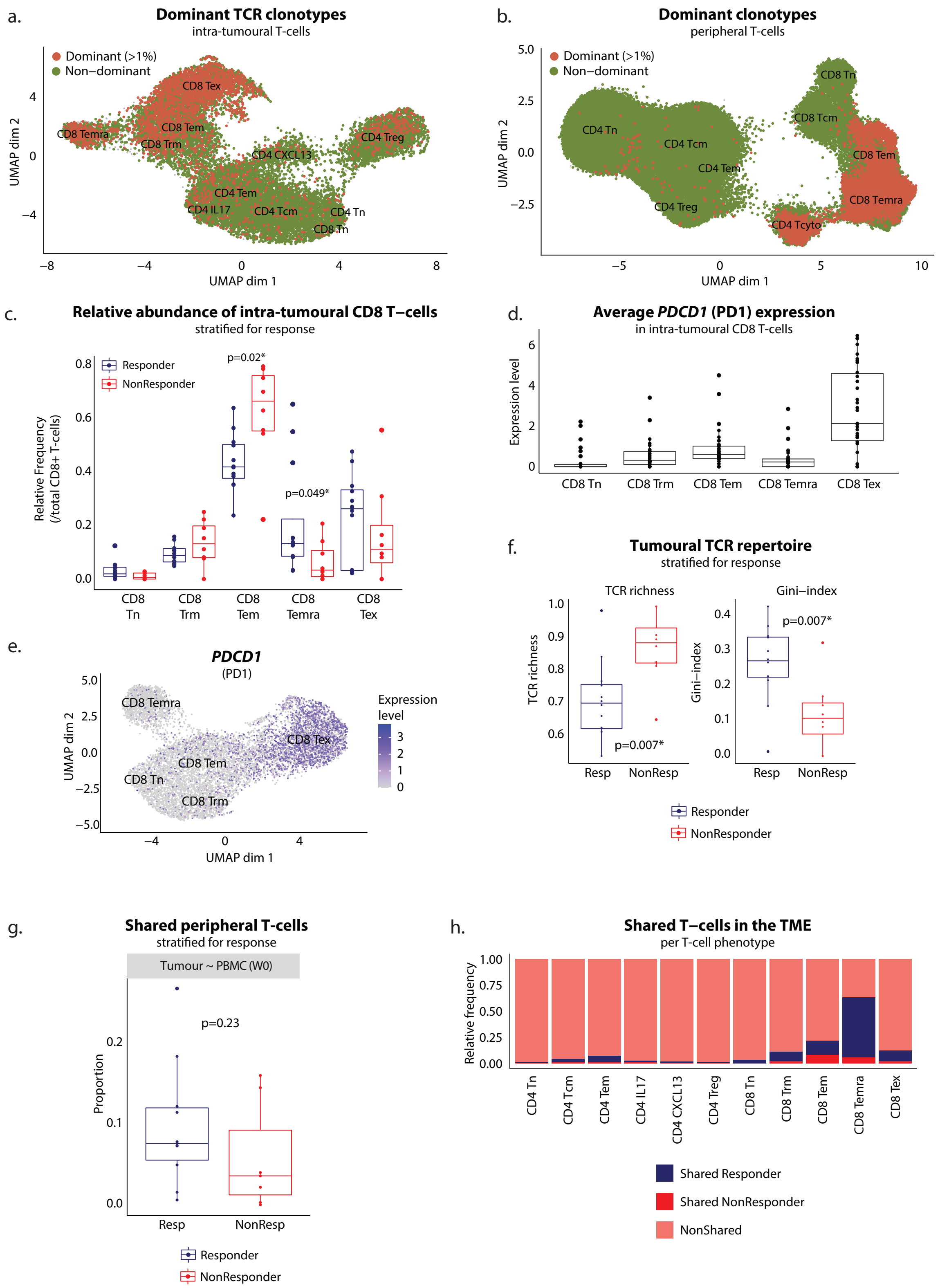

Supplementary Figure 3

Supplementary Figure 3. **Intra-tumoural T/NK-cells: comparative analyses**

a. UMAP representation of dominant versus non-dominant clonotypes in intra-tumoural T-cells. Dominant clonotypes were defined as clonotypes representing 1% or more of the TCR repertoire per sample. **b.** UMAP representation of dominant versus non-dominant clonotypes in peripheral T-cells. Dominant clonotypes were defined as clonotypes representing 1% or more of the TCR repertoire per sample. **c.** Boxplots depicting relative abundance of intra-tumoural CD8 T-cell phenotypes in atezo/bev-treated patients (n=20), calculated per patient and stratified for response. P-values calculated using two-sided Mann-Whitney U-test, only p-values <0.05 are shown. Boxes indicate median +/- interquartile range; whiskers show minima and maxima. **d.** Boxplots depicting average *PDCD1* (PD1) expression level, calculated per patient (n=38) in each intra-tumoural CD8 T -cell phenotype. **e.** Feature plot of *PDCD1* (PD1) expression in intra-tumoural CD8 T-cells. Boxes indicate median +/- interquartile range; whiskers show minima and maxima. **f.** TCR richness (*left*) and Gini-index (*right*) in intra-tumoural T-cells in atezo/bev-treated patients (n=20), calculated per patient and stratified for response. P-values calculated using two sample T-test. Boxes indicate median +/- interquartile range; whiskers show minima and maxima. **g.** Boxplot depicting the proportion of peripheral T-cells carrying TCRs shared between tumour and PBMCs at week 0, relative to the total number of T-cells detected in peripheral blood, calculated per sample (n=17) and stratified for response to atezo/bev. P-values calculated using two-sample T-test. Boxes indicate median +/- interquartile range; whiskers show minima and maxima. **h.** Stacked bar graph displaying the proportion of shared T-cells in responders and non-responders versus non-shared T-cells in the TME. Shared T-cells are characterized by a TCR found in peripheral blood prior to treatment (week 0).

(GD, Gamma-delta T-cells; PBMC, Peripheral blood mononuclear cells; PD1, Programmed cell death protein 1; TCR, T-cell receptor; TME, Tumour-microenvironment; UMAP, Uniform Manifold Approximation and Projection).

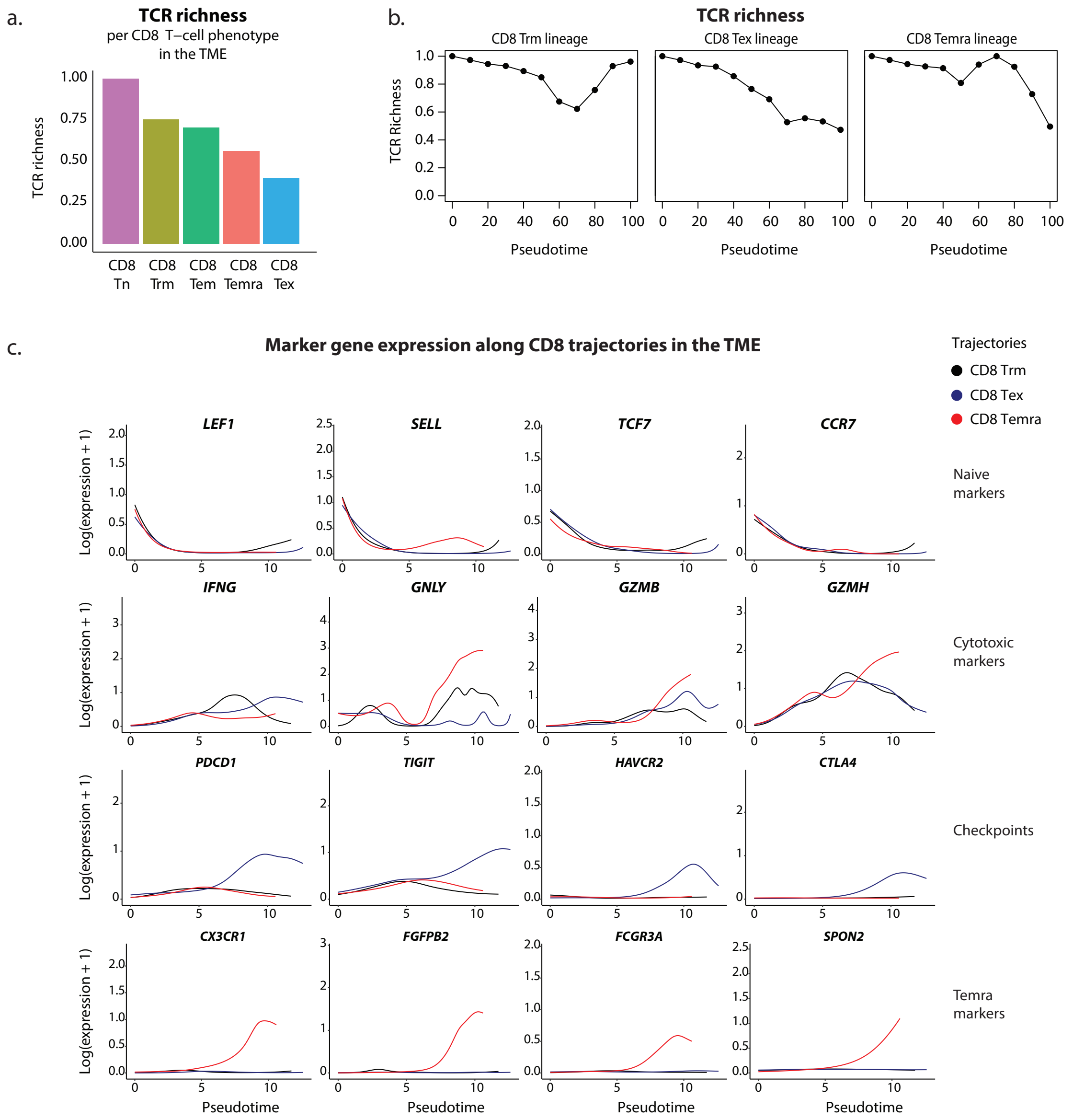

Supplementary Figure 4

Supplementary Figure 4. **Pseudo-time trajectories on intra-tumoural CD8 T-cells**

a. Bar plot showing TCR richness for each CD8 T-cell phenotype in the TME. **b.** TCR richness along each CD8 trajectory. **c.** Plots depicting the expression dynamics of marker and functional genes along each CD8 T-cell trajectory.

(TCR, T-cell receptor; TME, Tumour-microenvironment)

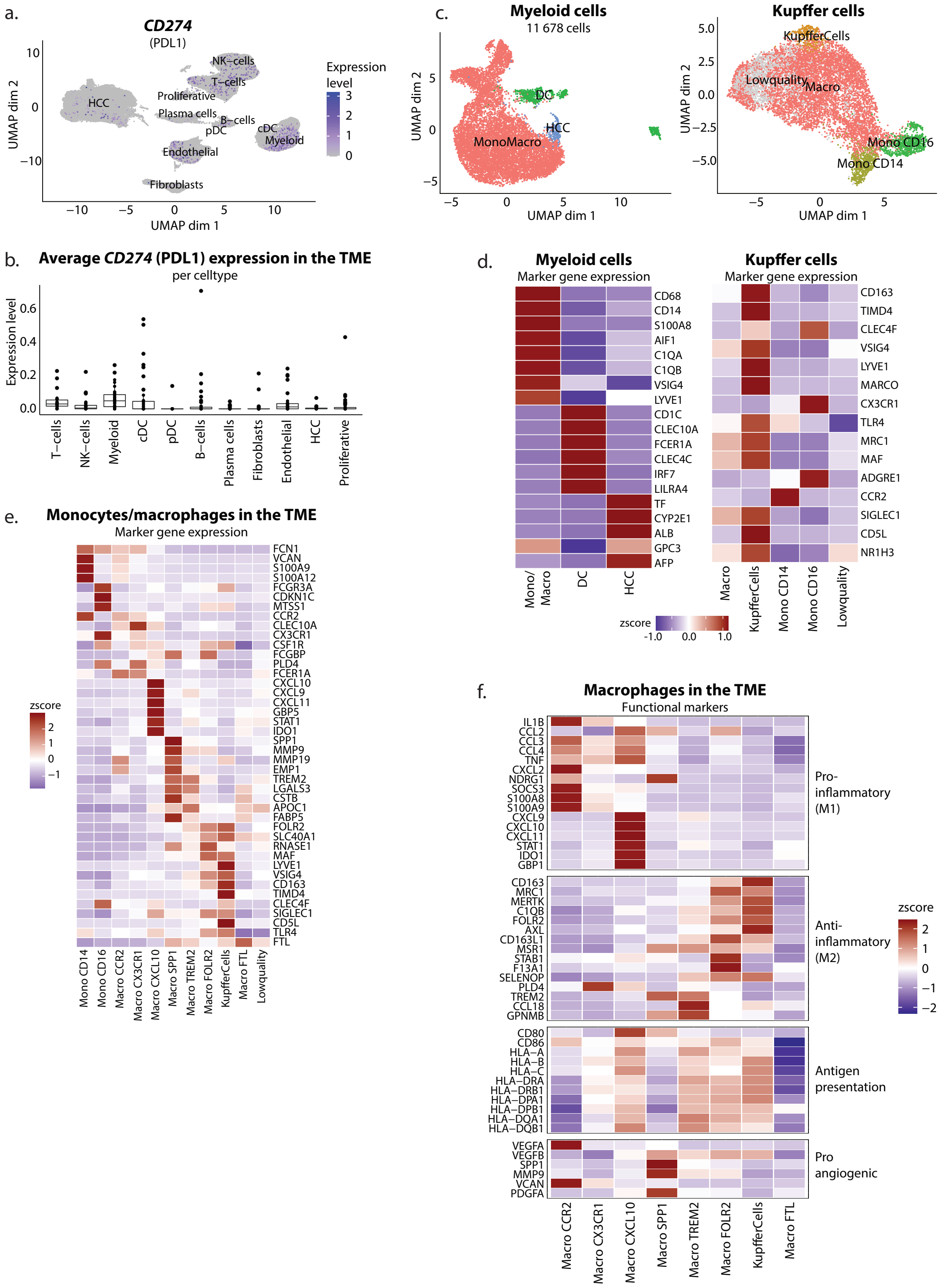

Supplementary Figure 5

Supplementary Figure 5. **Monocytes and macrophages in the TME of aHCC**

a. Feature plot of *CD274* (PDL1) expression in the TME. **b.** Boxplots depicting average *CD274* (PDL1) expression level per cell type in the TME, calculated per patient (n=38). Boxes indicate median +/- interquartile range; whiskers show minima and maxima. **c.** Subclustering of myeloid cells in the TME. *Left:* UMAP representation of myeloid cells in the TME identifying monocytes/macrophages and dendritic cells. *Right*: UMAP representation of macrophage subset in the TME, identifying Kupffer cells. **d.** *Left*: Heatmap displaying the expression of marker genes used for annotation of myeloid cells. *Right*: Heatmap displaying the expression of marker genes used for annotation of Kupffer cells. **e.** Heatmap displaying the expression of marker genes in each monocyte and macrophage phenotype. **f.** Heatmap depicting the expression of functional genes in macrophage subtypes.

(PDL1, Programmed death-ligand 1; TME, Tumour-microenvironment; UMAP, Uniform Manifold Approximation and Projection).

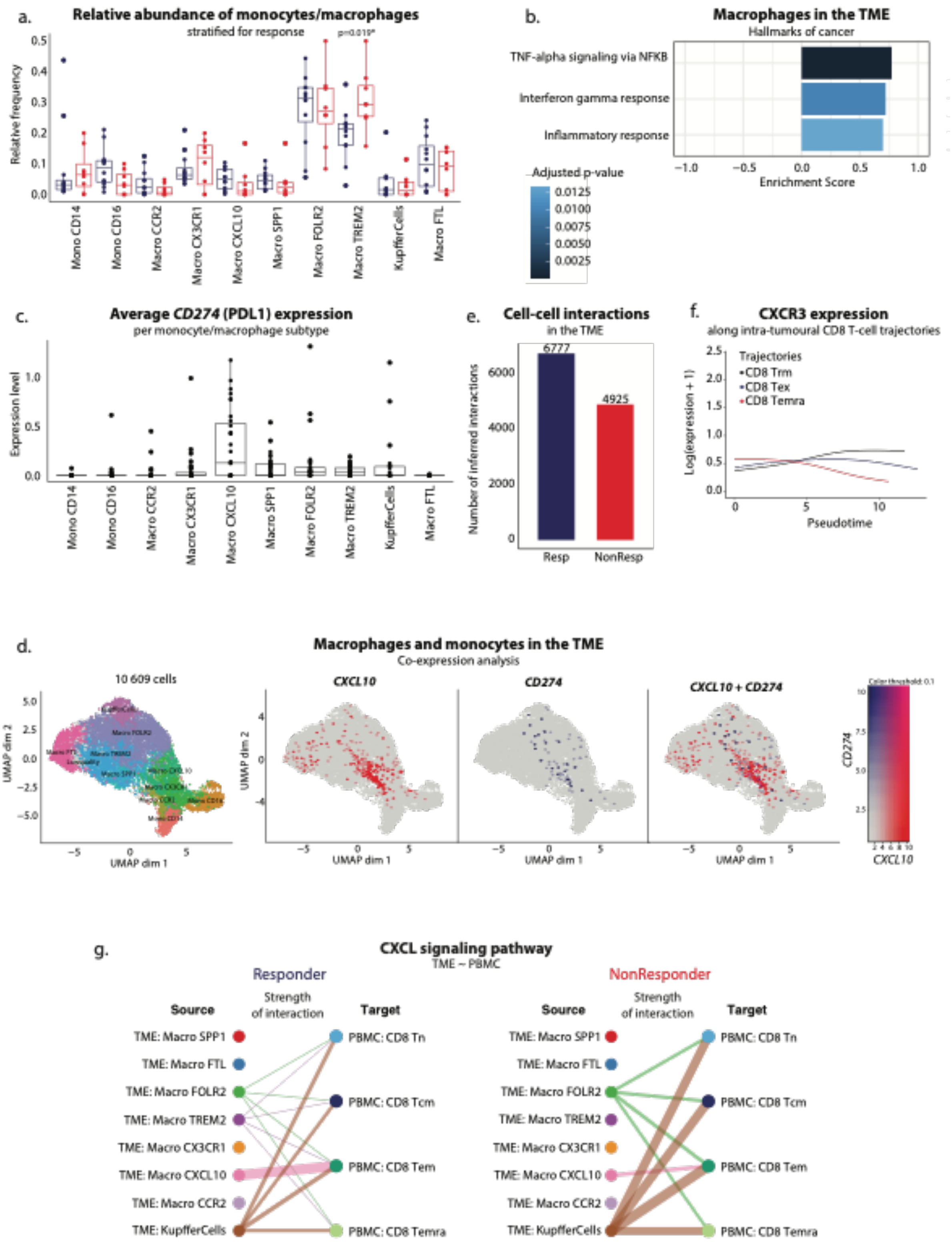

Supplementary Figure 6

Supplementary Figure 6. **Monocytes and macrophages: comparative analyses**

a. Boxplots depicting the relative abundance of monocyte and macrophage phenotypes in atezo/bev-treated patients (n=20), calculated per patient and stratified for response. P-values calculated using two-sided Mann-Whitney U-test, only p-values <0.05 are shown. Boxes indicate median +/- interquartile range; whiskers show minima and maxima. **b.** Pathway analysis on differentially expressed genes in macrophages in the TME of responders versus non-responders for the 'Hallmarks of cancer' gene sets. **c.** Boxplots depicting average *CD274* (PDL1) expression level, calculated per patient (n=38) in each monocyte and macrophage subtype. **d.** UMAP representation of intra-tumoural monocytes and macrophages, depicting the expression level of *CXCL10*, *CD274* and the combination of both. **e.** Bar plot showing the number of significant interactions between intra-tumoural myeloid cells and T-cells in responders versus non-responders. **f.** Plot depicting the expression dynamics of *CXCR3* along each CD8 T-cell trajectory within the TME. **g.** Hierarchy plot of the CXCL signaling pathway, depicting cell-cell interactions between intra-tumoural macrophages (source) and peripheral CD8 T-cells (target cells) in responders (*left)* and non-responders (*right)*. The width of edges represent the strength of communication.

(PDL1, Programmed death-ligand 1; TME, Tumour-microenvironment; UMAP, Uniform Manifold Approximation and Projection).

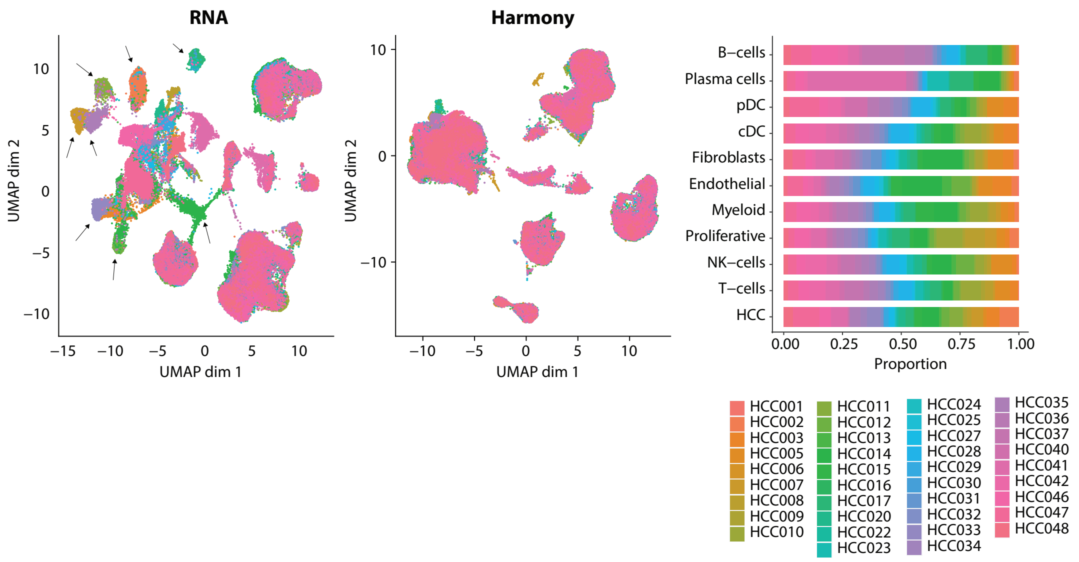

Supplementary Figure 7

Supplementary Figure 7. **Related to Methods**

UMAP colour-coded for individual patient with (*left)* and without *(middle)* integration using 'Harmony' and corresponding bar plot (*right)* showing the distribution of patients across cell types in the integrated dataset.

(UMAP, Uniform Manifold Approximation and Projection).

2. Supplementary Tables

Supplementary Table 1. **Patient demographics, tumour characteristics and treatment data**

	n=44
Age in years; median (range)	70 (24-85)
Male sex – no. (%)	34 (78)
Cirrhosis – no. (%)	26 (59)
Underlying liver disease	
Alcohol – no. (%)	18 (41)
Obesity-related fatty-liver disease – no. (%)	8 (18)
Viral hepatitis - no. (%)	3 (7)
Adenoma – no. (%)	1 (2)
Other – no. (%)	5 (11)
Unknown/None – no. (%)	9 (20)
Previous treatment for HCC – no. (%)	15 (34)
Liver transplantation – no. (%)	2 (5)
Local-regional – no. (%)	9 (20)
Systemic – no. (%)	2 (5)
TKI – no.	1
Chemotherapy – no.	1
Systemic + Local-regional – no. (%)	2 (5)
TKI + resection – no.	1
TKI + chemotherapy + resection – no.	1
AFP	
AFP in ng/mL – median (range)	8,70 (1,2-400800)
AFP >10 ng/mL – no. (%)	20 (45)
AFP >400 ng/mL – no. (%)	12 (27)
Child-Pugh Score	
Class A – no. (%)	39 (89)
Class B – no. (%)	5 (11)
Tumour type	
HCC – no. (%)	43 (98)
Mixed – no. (%)	1 (2)
Tumour characteristics at baseline	
BCLC C – no. (%)	26 (60)
Multinodular disease – no. (%)	35 (80)
Extrahepatic spread – no. (%)	14 (32)
Macrovascular invasion – no. (%)	14 (32)
Treatment	
Tyrosine-kinase inhibitor (TKI) – no. (%)	5 (11)
Sorafenib – no.	3

Lenvatinib – no.	2
Anti-PD(L)1 – no. (%)	11 (25)
Nivolumab, Pembrolizumab – no.	6
Atezolizumab – no.	5
Combination regimens	26 (59)
Atezolizumab/Bevacizumab - no.	25
Atezolizumab/Cabozantinib - no.	1
Untreated - no. (%)	2 (5)

HCC = Hepatocellular carcinoma; TKI = Tyrosine-kinase inhibitor; AFP = alpha-foetoprotein; Anti-PD(L)1 = antibody targeting PD1 (programmed cell death protein 1) or PDL1 (programmed death ligand 1))

Supplementary Table 2. **Overview of sample availability**

Patient ID	Tumour Biopsy	PBMC Week 0	PBMC Week 3	PBMC Week 6
HCC001	Biopsy	*NA*	*NA*	*NA*
HCC002	Biopsy	*NA*	*NA*	*NA*
HCC003	Biopsy	*NA*	*NA*	*NA*
HCC005	Biopsy	*NA*	*NA*	*NA*
HCC006	Biopsy	*NA*	*NA*	*NA*
HCC007	Biopsy	*NA*	*NA*	*NA*
HCC008	Biopsy	*NA*	*NA*	*NA*
HCC009	Biopsy	*NA*	*NA*	*NA*
HCC010	Biopsy	*NA*	*NA*	*NA*
HCC011	Biopsy	*NA*	*NA*	*NA*
HCC012	Biopsy	*NA*	*NA*	*NA*
HCC013	Biopsy	PBMC1	*NA*	PBMC3
HCC014	Biopsy 1&2*	PBMC1	PBMC2	PBMC3
HCC015	Biopsy	*NA*	*NA*	*NA*
HCC016	Biopsy	*NA*	*NA*	*NA*
HCC017	Biopsy	PBMC1	PBMC2	PBMC3
HCC020	Biopsy	PBMC1	PBMC2	PBMC3
HCC022	Biopsy	PBMC1	PBMC2	PBMC3
HCC023	Biopsy	PBMC1	PBMC2	PBMC3
HCC024	Biopsy	PBMC1	PBMC2	PBMC3
HCC025	Biopsy	PBMC1	PBMC2	PBMC3
HCC026	*NA*	PBMC1	PBMC2	PBMC3
HCC027	Biopsy	PBMC1	PBMC2	PBMC3
HCC028	Biopsy	PBMC1	PBMC2	PBMC3
HCC029	Biopsy	PBMC1	PBMC2	PBMC3
HCC030	Biopsy	PBMC1	PBMC2	PBMC3

HCC031	Biopsy	PBMC1	PBMC2	*NA*
HCC032	Biopsy 1&2*	*NA*	*NA*	*NA*
HCC033	Biopsy	*NA*	*NA*	*NA*
HCC034	Biopsy	PBMC1	PBMC2	PBMC3
HCC035	Biopsy	*NA*	*NA*	*NA*
HCC036	Biopsy	PBMC1	PBMC2	PBMC3
HCC037	Biopsy	*NA*	*NA*	*NA*
HCC040	Biopsy	PBMC1	PBMC2	PBMC3
HCC041	Biopsy	*NA*	*NA*	*NA*
HCC042	Biopsy	PBMC1	PBMC2	PBMC3
HCC046	Biopsy	PBMC1	PBMC2	PBMC3
HCC047	Biopsy	PBMC1	PBMC2	*NA*
HCC048	Biopsy	*NA*	*NA*	*NA*
HCCX4	*NA*	PBMC1	PBMC2	PBMC3
HCCX5	*NA*	PBMC1	PBMC2	PBMC3
HCCX6	*NA*	PBMC1	PBMC2	PBMC3
HCCX7	*NA*	PBMC1	PBMC2	PBMC3
HCCX8	*NA*	PBMC1	PBMC2	PBMC3

PBMC=peripheral mononuclear cells; NA = not available.
**Two biopsies were obtained from the same tumour nodule.*

Supplementary Table 3. **PBMC pooling matrix**

ID	Sample 1	Sample 2	Sample 3
PBMC 1	HCC013 – W0	HCCX4 – W0	
PBMC 2	HCC013 – W6	HCCX7 – W0	
PBMC 3	HCC014 – W0	HCCX4 – W3	
PBMC 4	HCC014 – W3	HCCX5 – W0	
PBMC 5	HCC014 – W6	HCCX5 – W3	
PBMC 6	*test samples*	HCCX5 – W6	
PBMC 7	*test samples*	HCCX4 – W6	
PBMC 8	*test samples*	HCCX7 – W3	
PBMC 9	HCC017 – W0	HCC023 – W6	
PBMC 10	HCC017 – W3	HCCX6 – W0	
PBMC 11	HCC017 – W6	HCCX6 – W3	
PBMC 12	HCC020 – W0	HCCX6 – W6	

PBMC 13	HCC020 – W3	HCCX7 – W6	
PBMC 14	HCC020 – W6	HCCX8 – W0	
PBMC 15	HCC023 – W0	HCCX8 – W3	
PBMC 16	HCC023 – W3	HCCX8 – W6	
PBMC 17	*test samples*	HCC031 – W0*	
PBMC 18	HCC024 – W0	*test samples*	
PBMC 19	HCC024 – W3	HCC025 – W6	
PBMC 20	HCC024 – W6	HCC029 – W0	
PBMC 21	HCC028 – W0	HCC026 – W0	
PBMC 22	HCC028 – W3	HCC026 – W3	
PBMC 23	HCC028 – W6	HCC029 – W3	
PBMC 24	HCC025 – W0	HCC029 – W6	
PBMC 25	HCC025 – W3	HCC026 – W6	
PBMC 26	HCC022 – W0	HCC031 – W3	
PBMC 27	HCC022 – W3	HCC034 – W0	
PBMC 28	HCC022 – W6	HCC034 – W3	
PBMC 29B	HCC030 – W0	HCC034 – W6	
PBMC 30	HCC030 – W3	HCC036 – W0	
PBMC 31	HCC030 – W6	HCC036 – W3	
PBMC 32	HCC031 – W0*	HCC036 – W6	
PBMC 42	HCC042 – W0	HCC040 – W0	HCC047 – W0
PBMC 43	HCC043 – W3	HCC046 – W3	HCC027 – W6
PBMC 44	HCC042 – W6	HCC040 – W6	HCC046 – W0
PBMC 45	HCC040 – W3	HCC046 – W6	HCC027 – W0
PBMC 46	HCC047 – W3	HCC027 – W3	

same sample
