# Supplementary material for: PD-1- CD45RA+ effector-memory CD8 T cells and CXCL10+ macrophages are associated with response to atezolizumab plus bevacizumab in advanced hepatocellular carcinoma
Source: Nat Commun. 2023 Nov 29;14:7825. doi: 10.1038/s41467-023-43381-1 (PMC10687033; doi:10.1038/s41467-023-43381-1)
Supplement: Supplementary file 3 — Description of additional supplementary files [file 41467_2023_43381_MOESM3_ESM.pdf]

<u>Description of additional supplementary files</u>

Supplementary Dataset 1: Overview of 162 antibodies from TotalSeq-C (Biolegend)
