# Supplementary material for: PD-1- CD45RA+ effector-memory CD8 T cells and CXCL10+ macrophages are associated with response to atezolizumab plus bevacizumab in advanced hepatocellular carcinoma
Source: Nat Commun. 2023 Nov 29;14:7825. doi: 10.1038/s41467-023-43381-1 (PMC10687033; doi:10.1038/s41467-023-43381-1)
Supplement: Supplementary file 5 — Reporting Summary [file 41467_2023_43381_MOESM5_ESM.pdf]

nature portfolio

Corresponding author(s): Jeroen Dekervel

Last updated by author(s): Sep 12, 2023

Reporting Summary

Nature Portfolio wishes to improve the reproducibility of the work that we publish. This form provides structure for consistency and transparency in reporting. For further information on Nature Portfolio policies, see our Editorial Policies and the Editorial Policy Checklist.

Statistics

For all statistical analyses, confirm that the following items are present in the figure legend, table legend, main text, or Methods section.

n/a	Confirmed	
☐	☒	The exact sample size (*n*) for each experimental group/condition, given as a discrete number and unit of measurement
☐	☒	A statement on whether measurements were taken from distinct samples or whether the same sample was measured repeatedly
☐	☒	The statistical test(s) used AND whether they are one- or two-sided<br>*Only common tests should be described solely by name; describe more complex techniques in the Methods section.*
☐	☒	A description of all covariates tested
☐	☒	A description of any assumptions or corrections, such as tests of normality and adjustment for multiple comparisons
☐	☒	A full description of the statistical parameters including central tendency (e.g. means) or other basic estimates (e.g. regression coefficient) AND variation (e.g. standard deviation) or associated estimates of uncertainty (e.g. confidence intervals)
☐	☒	For null hypothesis testing, the test statistic (e.g. *F*, *t*, *r*) with confidence intervals, effect sizes, degrees of freedom and *P* value noted<br>*Give P values as exact values whenever suitable.*
☒	☐	For Bayesian analysis, information on the choice of priors and Markov chain Monte Carlo settings
☒	☐	For hierarchical and complex designs, identification of the appropriate level for tests and full reporting of outcomes
☐	☒	Estimates of effect sizes (e.g. Cohen's *d*, Pearson's *r*), indicating how they were calculated

Our web collection on statistics for biologists contains articles on many of the points above.

Software and code

Policy information about availability of computer code

Data collection	Single-cell RNA sequencing reads were mapped to the GRCh38 human reference genome and processed into gene expression matrices using CellRanger (10x Genomics; version 3.1.0). TCR (V(D)J) alignment and annotation was achieved using CellRanger VDJ (10x Genomics; version 3.1.0).

Data analysis	Single-cell RNA sequencing data was analysed using Seurat (version 4; Hao et al Cell 2021). PBMC samples were pooled and and assigned back to their sample of origin using the Souporcell tool (Heaton H et al. Nat Methods 2020). Copy number variations were assessed with the R package inferCNV (version 1.2.1; Tickle et al 2019). Trajectory inference analysis was performed using the R package Slingshot v2.2.0 (Street K et al BMC Genomics 2018) and TradeSeq (version 1.7.07; Van den Berge K et al Nat Commun 2020) was used to identify differentially expressed genes between trajectories. Cell-cell interaction analysis was performed using CellChat (version 1.1.3; Jin S et al Nat Commun 2021). TCR Gini-index was calculated using the ineq (version 0.2-13) R package.<br>Differentially expressed genes were identified using Wilcoxon's test with the FindMarkers function from Seurat (version 4). ClusterProfiler (version 4.055) was used to perform Over-Representation-Analysis for the 'Hallmarks of cancer' gene set from MSigDB (version 6.2).<br>Survival analysis was done using the R packages 'survival' (version 3.3.1) and 'survminer' (version 0.4.9). The R package 'maxstat' (version 0.7.25) was used to delineate groups (biomarker high versus biomarker low) using maximally selected rank statistics.<br><br>Additionally, a publicly available bulk RNAseq dataset was accessed (see Data section below for details). The raw read files were mapped to the human reference genome (refdata-gex-GRCh38-2020-A) using the STAR aligner (STAR.2.7.2a) in paired-end mode. Gene counts per sample were then computed using featureCounts (Subread toolkit) and the RNA counts were normalized based on the trimmed mean of M-values (TMM) method using the R-package edgeR (version 3.3.2). The per sample gene set enrichment scores were calculated using the R-package 'GSVA' (version 1.38.2).

For manuscripts utilizing custom algorithms or software that are central to the research but not yet described in published literature, software must be made available to editors and reviewers. We strongly encourage code deposition in a community repository (e.g. GitHub). See the Nature Portfolio guidelines for submitting code & software for further information.

Data

Policy information about availability of data

All manuscripts must include a data availability statement. This statement should provide the following information, where applicable:

- Accession codes, unique identifiers, or web links for publicly available datasets
- A description of any restrictions on data availability
- For clinical datasets or third party data, please ensure that the statement adheres to our policy

Raw sequencing reads of the scRNAseq, scTCRseq and Totalseq experiments will be deposited under restricted access in the European Genome-phenome Archive (EGAS00001007547) upon publication. Requests for accessing raw sequencing reads will be reviewed by the UZLeuven-VIB data access committee. Any data shared will be released via a Data Transfer Agreement that will include the necessary conditions to guarantee protection of personal data (according to European GDPR law). Alternatively, a download of the read count data per sample, necessary to reproduce all analyses included in this manuscript, will be made available at https://lambrechtslab.sites.vib.be/en/data-access. Source data are provided with this paper.

The per sample raw RNA read files from the publicly available bulk RNAseq dataset and associated clinical data were downloaded from the European Genome Archive (EGAS0001005503; DA00468).

Research involving human participants, their data, or biological material

Policy information about studies with human participants or human data. See also policy information about sex, gender (identity/presentation), and sexual orientation and race, ethnicity and racism.

Reporting on sex and gender	Data concerning patient demographics (including sex) were collected at time of inclusion. All patient demographics are reported as aggregated data in Supplementary Table 1. As expected in a typical HCC population, the majority of the patients were male.
Reporting on race, ethnicity, or other socially relevant groupings	Race and ethnicity are not reported in our manuscript.
Population characteristics	All advanced HCC patients eligible for systemic therapy at the University Hospitals Leuven were invited to participate in our study. Patient demographics prior to treatment were recorded, including sex, age, the presence of cirrhosis and type of underlying liver disease. Baseline serum alpha-foetoprotein levels and liver function based on the Child-Pugh Score were also recorded. Baseline tumour characteristics including tumour type and the presence for macrovascular invasion and/or extrahepatic disease were noted. Information relating to treatment including treatment type, treatment response and clinical outcome were collected. Radiological response was evaluated by computed tomography (CT) or magnetic resonance imaging (MRI) approximately every 3 months, according to standard clinical practice and assessed by an independent radiologist using the modified RECIST criteria. Response was defined as objective response (partial or complete response) at 3 months or disease control (stable disease) during at least 6 months after treatment initiation.<br><br>Potential sources of selection bias:<br>- The evolution of the treatment landscape of advanced HCC since the start of the study from TKI's to immunotherapy-based combination regimens, is reflected in the number of patients in each treatment group. This resulted in a small number of patients treated with TKI's.
Recruitment	Between December 2018 and June 2023, all patients diagnosed with advanced HCC and eligible for systemic treatment at the University Hospitals Leuven, were invited to participate in our study. Clinical eligibility was based on good performance status (ECOG 0-1) and adequate hematologic and end-organ function. If deemed eligible, they were recruited into the study. Selection of systemic treatment was at the discretion of the treating physician, guided by clinical practice guidelines, individual patient eligibility and treatment availability at time of inclusion.

	Potential sources of bias:<br>- This was a single-center, prospective, non-randomized, observational study. Treatment selection was at the discretion of the treating physician based on individual patient eligibility and treatment availability at the time of inclusion which results in a selection bias.<br>- Considering the limited number of events in the scRNAseq cohort, the survival analyses were not corrected for baseline patient or tumour characteristics in a multivariate analysis.
Ethics oversight	The study was approved by the Ethics Committee of University Hospitals Leuven (UZ/KUL, S62548). All patients gave written informed consent for the use of their samples for research purposes.

Note that full information on the approval of the study protocol must also be provided in the manuscript.

Field-specific reporting

Please select the one below that is the best fit for your research. If you are not sure, read the appropriate sections before making your selection.

[x] Life sciences [ ] Behavioural & social sciences [ ] Ecological, evolutionary & environmental sciences

For a reference copy of the document with all sections, see nature.com/documents/nr-reporting-summary-flat.pdf

Life sciences study design

All studies must disclose on these points even when the disclosure is negative.

Sample size	This was a single-center, prospective, non-randomized translational study. Using single-cell sequencing technology, we aimed to characterize the intra-tumoural and peripheral immune system of advanced HCC patients prior to and during systemic treatment. Overall, 44 advanced HCC patients were included. Selection of systemic treatment was at the discretion of the treating physician, guided by clinical practice guidelines, individual patient eligibility and treatment availability at time of inclusion. Prospective sample collection included a fresh tissue biopsy before start of treatment and serial PBMC samples collected prior to and during treatment (week 0-3-6). Overall 38 pre-treatment tissue biopsies and 72 serial PBMC samples were collected. An overview of available samples is provided in Supplementary Table 2. All samples were subjected to simultaneous scRNAseq and scTCRseq. Additional epitope profiling was performed by TotalSeq-C (Biolegend) on a subset of PBMC samples (n=41).<br><br>scRNAseq data from all available samples was used for clustering and annotation of single cells into their respective tumoural and peripheral cell (pheno-)types. As we specifically aimed to explore the effect of treatment with atezolizumab plus bevacizumab all subsequent comparative analyses focused on patients treated with atezolizumab plus bevacizumab (n=25). Radiological response was evaluated by computed tomography (CT) or magnetic resonance imaging (MRI) approximately every 3 months, according to standard clinical practice and assessed by an independent radiologist using the modified RECIST criteria. Response was defined as objective response (partial or complete response) at 3 months or disease control (stable disease) during at least 6 months after treatment initiation. Various single-cell readouts, including therapeutic target expression, differential gene expression, TCR metrics were compared between responders and non-responders.
Data exclusions	No scRNAseq samples were excluded. One sample was excluded from the TCR analysis due to a low number of detected TCR.
Replication	Not applicable as no technical replicates were used, only biological replicates. Due to the high cost of scRNAseq, the inherent risk of liver biopsies in a fragile HCC patients and the scarcity of tumor tissue obtained by needle biopsies as well as the fact that it is unethical to take an unlimited number of needle biopsies from liver cancer patients, we could not include technical replicates for single-cell analyses.
Randomization	Patients were not randomized in this study. Selection of systemic treatment was at the discretion of the treating physician, guided by clinical practice guidelines, individual patient eligibility and treatment availability at time of inclusion. Indeed, the distribution of patients across treatment groups reflects the evolution of the treatment landscape of advanced HCC since the start of this study from TKI's to immunotherapy-based combinations.
Blinding	This was a single-center, prospective, non-randomized translational study, therefore there was no blinding in this study.

Reporting for specific materials, systems and methods

We require information from authors about some types of materials, experimental systems and methods used in many studies. Here, indicate whether each material, system or method listed is relevant to your study. If you are not sure if a list item applies to your research, read the appropriate section before selecting a response.

Materials & experimental systems

n/a	Involved in the study
☐	☒ Antibodies
☒	☐ Eukaryotic cell lines
☒	☐ Palaeontology and archaeology
☒	☐ Animals and other organisms
☒	☐ Clinical data
☒	☐ Dual use research of concern
☒	☐ Plants

Methods

n/a	Involved in the study
☒	☐ ChIP-seq
☒	☐ Flow cytometry
☒	☐ MRI-based neuroimaging

Antibodies

Antibodies used	Epitope profiling was performed by TotalSeq-C (Biolegend) on 41 PBMC samples. 162 oligo-conjugated antibodies (1:1000 diluted in labeling buffer) from the TotalSeq-C panel. This panel is commercially available and distributed by BioLegend (TotalSeq™-C Human Universal Cocktail, V1.0). The panel has been designed to react with 130 unique cell surface antigens, including principal lineage antigens, and includes 7 isotype control antibodies, to aid in the multi-omic characterization of immune cells. The antibodies in the cocktail are provided at optimized concentrations to provide a ready-to-use solution once the cocktail has been reconstituted.<br><br>Further details, including the full list of antibodies can be found on the TotalSeq™-C Human Universal Cocktail product weblink: https://www.biolegend.com/ja-jp/products/totalseq-c-human-universal-cocktail-v1-0-19736?GroupID=GROUP28.<br>A full list of antibodies used is provided as Supplementary Dataset 1.
Validation	All TotalSeq-C antibody lots are tested for Proteogenomics (i.e., Antibody oligonucleotide conjugates designed to enable the generation of transcriptomic and proteomic data simultaneously, using single-cell devices such as 10x Genomics' Chromium and Illumina sequencers) by immunofluorescent staining using flow cytometric analysis. The attached oligomer sequence is confirmed by sequencing. More details can be found at https://www.biolegend.com/en-us/quality/quality-control and https://www.biolegend.com/en-us/quality/reproducibility.
